# A carbon monoxide cycle drives carbon monoxide uptake and poisoning

**DOI:** 10.14814/phy2.70858

**Published:** 2026-04-15

**Authors:** Ronald F. Coburn, Michael S. Tift

**Affiliations:** ^1^ Department of Physiology, The Perelman School of Medicine The University of Pennsylvania Philadelphia Pennsylvania USA; ^2^ Department of Biology and Marine Biology University of North Carolina Wilmington Wilmington North Carolina USA

**Keywords:** carbon monoxide poisoning, carbon monoxide reaction with oxyhemoglobin, carbon monoxide uptake, generation of tissue P_CO_, R and T allosteric hemoglobin states

## Abstract

An understanding of the physiology of acute carbon monoxide (CO) poisoning remains incomplete. This study describes a novel approach—considering a CO cycle driven by CO inhalation which includes: alveolar CO uptake → the transport to peripheral tissues → an increase in the P_CO_ and [COHb] in peripheral capillary blood → and a return of COHb to the lungs. Unlike earlier models, this model allows evaluation of how [COHb] changes will affect physiological events at different sites in this cycle. We calculated increases in the P_CO_ and the [COHb] at these sites during constant breathing of different CO concentrations, using an approach that emphasizes the importance of the rate of the replacement reaction (CO + oxyhemoglobin (O_2_Hb) ↔ COHb + oxygen (O_2_)) in the physiology of CO poisoning. Key findings include: (i) how interactions between inhaled CO, COHb recirculating back to alveolar capillaries, and alveolar capillary P_CO_ back‐pressure regulate pulmonary CO uptake; (ii) how a decrease in the arterial [O_2_Hb] evokes an amplification of the P_CO_ in blood entering peripheral tissues; (iii) that hemoglobin's R‐to‐T allosteric shifts influence CO delivery to peripheral tissues; and (iv) a clearer characterization of how tissue P_CO_ is increased during CO exposures.

## 1 INTRODUCTION

A previous study (Coburn, 2018) described how increases in the partial pressure of carbon monoxide (P_CO_) can be generated in peripheral tissues during acute carbon monoxide (CO) poisoning and how increases in the carboxyhemoglobin % saturation ([COHb]) in blood flowing from the lungs to these tissues are transduced to increases in their mean extravascular tissue P_CO_ (mTiss‐P_CO_). An increased mTiss‐P_CO_ evokes CO binding to ferroproteins which can cause toxic effects (reviewed by Coburn, 2022). Relationships of the [COHb] entering peripheral tissues (Entry‐[COHb]) to their mTiss‐P_CO_ described in this previous article were dependent on concepts described by the Coburn Forster Kane Equation (Coburn et al., 1965), a publication that verified this equation for low CO exposures (Peterson & Stewart, 1970), and reports that describe the kinetics of the replacement reaction (ReplR); (CO + oxyhemoglobin (O_2_Hb) ↔ COHb + oxygen (O_2_)) (Holland, 1969a, 1970; Roughton, 1964). However, our understanding of the physiology of peripheral capillary P_CO_ (mPC‐P_CO_) and mTiss‐P_CO_ increases in different tissues during the CO uptake phase of acute CO poisoning is still incomplete. While the importance of alveolar capillary mean P_CO_ back‐pressure (BKP‐P_CO_) in regulating pulmonary CO uptake and the lung's steady‐state CO diffusing capacity (DL_CO_) are well established (Hughes & Bates, 2003), there has been no prior analysis on how pulmonary CO uptake interacts with BKP‐P_CO_ across varying levels of [COHb] entering the alveolar capillaries (Inlet‐[COHb]) during constant inhalation of different CO concentrations. Additionally, no previous analysis has examined how decreases in the [O_2_Hb] resulting from increases in the [COHb] can result in amplifying the P_CO_ in blood arriving to peripheral tissues (Entry‐P_CO_). While the involvement of a hemoglobin (Hb) R‐to‐T state transitions could impact rates of CO pulmonary uptake and delivery to peripheral tissues, this has also not been well‐studied. Although, as indicated above, the mechanism by which increased [COHb] in peripheral capillary blood leads to an elevated P_CO_ has been described, a comprehensive analysis of all contributing factors has not been conducted.

In this study, we present a new approach for understanding or predicting how inhaled CO is distributed across different body regions, using a cycle‐based model, along with analyses and calculations that clarify key aspects of this process. Our goal is to determine the physiological variables that regulate increases in P_CO_ at the different sites in the CO cycle during constant CO breathing in resting adult male humans. This will allow readers to obtain a better understanding of the physiology of CO poisoning. Previous models (Coburn, 2018) considered the timing of increases in the [COHb] that occurs during the breathing of different [CO] determined by rates of alveolar CO uptake and the extent of total body blood and extravascular CO hemoprotein binding. The model described in the present manuscript analyzes events that occur at different sites in the CO cycle.

**FIGURE 1:**
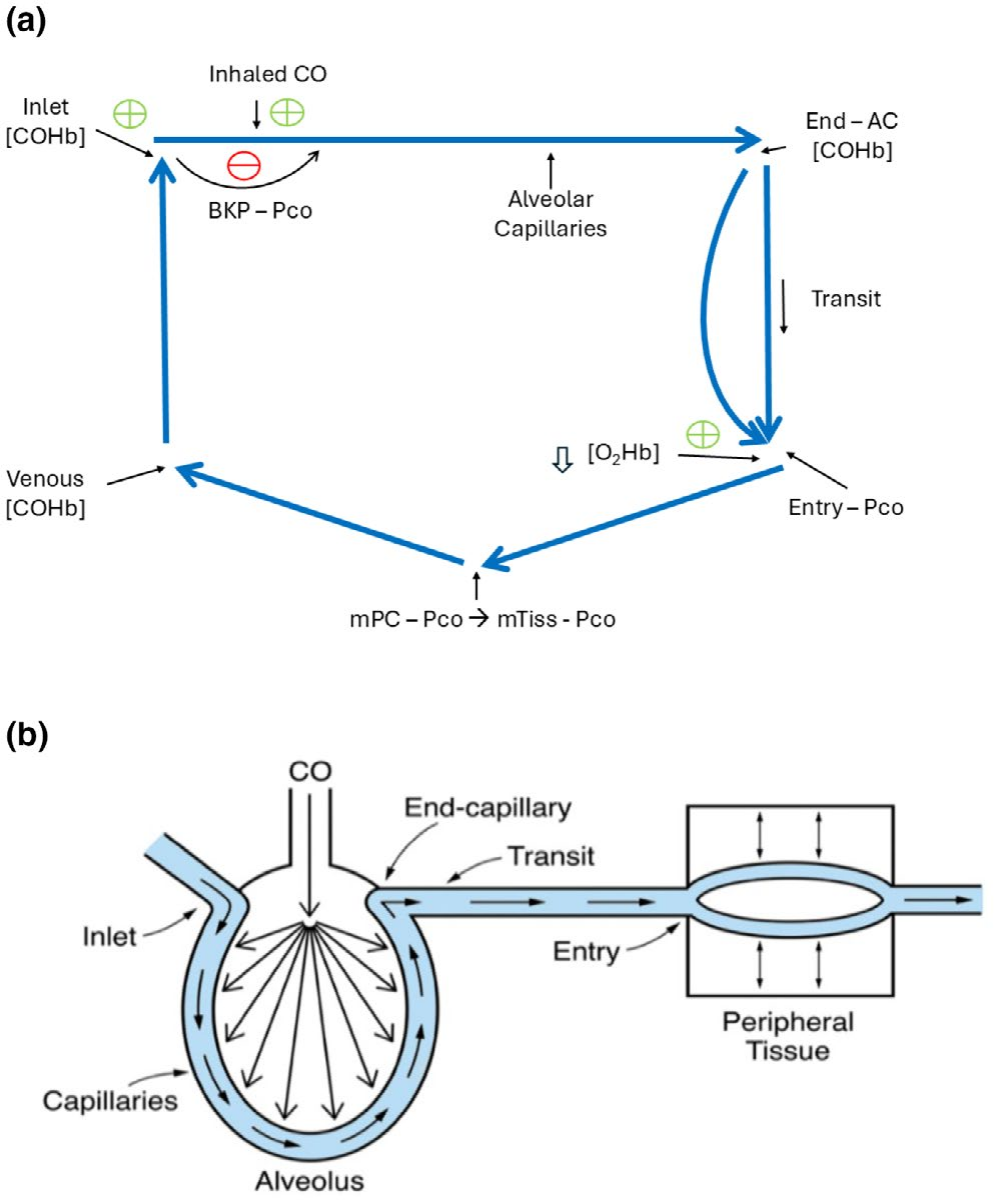
(a) The CO cycle during constant CO breathing. Abbreviations used in this manuscript not defined above are—↑ AC[COHb], the increase in the [COHb] due to CO entering alveolar capillaries; the End‐AC[COHb], the [COHb] in blood exiting alveolar capillaries. (b) Alveolar capillary CO uptake, CO transit, and peripheral tissue CO uptake. “Transit” indicates blood flowing from end‐alveolar capillaries via pulmonary veins, the left atrium and ventricle to peripheral tissues. Arrows within “Peripheral Tissue” depict a likely steady‐state CO diffusion equilibrium between capillary blood and extravascular tissue that is discussed later in this article.

The CO cycle is depicted in Figure 1a. The uptake of CO into alveolar capillaries and transport to and within peripheral tissues involved in this cycle are illustrated in Figure 1b.

As cited above, previous articles documented the importance of the kinetics of the ReplR that enhanced an understanding of the physiology of pulmonary capillary CO uptake and the DL_CO_ (Chakraborty et al., 2004; Coburn et al., 1965; Forster, 1964; Roughton & Forster, 1957). In studies of human erythrocyte suspensions performed at 37°C and pH 7.40, this reaction was shown to have a T_1/2_ of 41 msecs (Holland, 1969a, 1970; This was measured using a stop‐flow apparatus. Association velocity constants measured using this method are probably slightly underestimated due to diffusion limitation and the T_1/2_ may be even shorter than 41 msec). Results of measurements of [COHb] increases in blood exiting dog lungs after a rapid CO inhalation illustrate the reaction's importance during CO uptake into alveolar capillary blood and also indicated that chemical equilibrium (Chem Eq) in terms of the ReplR has not occurred in blood exiting alveolar capillaries (Abboud et al., 1974; In this experiment where aortic blood was sampled every sec, a rapid inhalation of 5% CO from the resting lung volume to nearly the total lung capacity resulted in a [COHb] increase of 51% sat. after 2 secs of breath holding). Thus, this reaction continues in blood while flowing towards peripheral tissues (Coburn, 2018). The transit time from alveolar capillaries through the pulmonary veins, heart, and arterial system to microcirculations such as the coronary and cerebral beds can be as short as 2 s, equivalent to approximately 50 half‐times (T_1/2_) of the ReplR. The mixing that occurs during transit should minimize diffusion limitation of this reaction. Thus, there is compelling evidence that the P_CO_ in blood arriving at the Entry (Entry‐P_CO_) is in a near Chem Eq with terms of the ReplR (Complete Chem Eq never occurs and the term used in this article indicates a near Chem Eq).

Results of calculations performed in a previous study (Coburn, 2018) indicate the reaction rate of the ReplR in blood flowing within some peripheral tissue capillaries may be rapid enough to reach a Chem Eq, a topic discussed later in this article. The equilibrium constant of the ReplR, M, which describes relative Hb binding affinities of CO and O_2_, has been used to estimate increases in the mPC‐P_CO_ during CO inhalations (Coburn, 2018). According to Haldane's laws, M is a constant (Haldane & Smith, 1896). However, this is true only when Hb is fully saturated. Decreases in binding of ligands to tetrameric Hb evoke an allosteric transition from a relaxed (R) to a tense (T) state (Ahmed et al., 2020), which results in a decrease in the CO affinity for Hb binding (Di Cera et al., 1987). The reported values of M are 241 ± 29 in the R state (M_R_) and 123 ± 34 in the T state (M_T_) (Di Cera et al., 1987). The Bohr shift evoked by decreasing pH promotes the R‐to‐T state transition and thus stabilizes the T state (Ahmed et al., 2020). Rodkey et al. (1969) reported that under conditions of full Hb saturation, in both Hb and erythrocyte solutions, the relative binding affinities of CO and O_2_ were consistent across over 100‐fold changes in CO/O_2_ ratios, regardless of pH (6.8–8.8) and the presence or absence of CO_2_. Although there is still debate about how quickly administered CO equilibrates between capillary blood and extravascular tissues (reviewed by Bruce et al., 2008), several lines of evidence suggest the process is rapid: (a) findings obtained in dog experiments that mixing of administered CO in vascular plus extravascular stores occurs at the same rate as mixing of injected erythrocytes within the vascular circulation (Luomanmaki & Coburn, 1969); (b) the high affinity and rapid association of CO with many extravascular hemoproteins (Collman et al., 1976); (c) and observations of rapid CO transfers from capillary blood to extravascular tissues in the heart and skeletal muscle during arterial hypoxemia or reduced blood flow (Coburn, 2021).

## 2 METHODS

We calculated increases in the P_CO_ at the different locations of the CO cycle during constant breathing of CO by a resting normal human of 0.1% (1000 ppm), 0.3% (3000 ppm), or 1.0% (10,000 ppm), concentrations that can evoke clinical poisoning symptoms and death (Greiner, 1997). Single cycle data were calculated using different Inlet‐[COHb] achieved during constant breathing of the three different CO concentrations. Constants used in these calculations were the steady state DL_CO_, a cardiac output, the BKP‐P_CO_—different calculated values; an alveolar PO_2_ of 100 mmHg; the PO_2_ at peripheral tissue Entry sites of 100 mmHg; a [Hb] of 15 g/100 mL; M_R_ of 241; M_T_ of 123. These constants are relevant to healthy young men. Calculations followed established findings that the minute ventilation and cardiac output do not increase as the [COHb] increases to 30 to 35% sat. levels (Asmussen & Chiodi, 1941; Ayres et al., 1970; Chiodi et al., 1941; Penney, 1988). In addition, our calculations considered that significant changes in the [COHb] do not occur as blood flows from the lung to Entry sites, and then to the Inlet, even though, as described later in this article, there are decreases in the blood P_CO_. The reason for this is the low solubility of dissolved CO in blood (0.0023 mL/100 mL blood × mmHg at 38°C (Power, 1968)). This indicates that a blood P_CO_ decrease of 1 mmHg would result in less than a 0.01% COHb increase mediated by operation of the ReplR. Assumptions made in the calculations are; that the alveolar PO_2_ does not change as the [COHb] increases; that the ReplR becomes activated due to decreasing PO_2_ levels that occur as blood flows within peripheral capillaries; that the ReplR is rapid enough so that Chem Eq is achieved in blood flowing within peripheral capillaries; that a transition of Hb from the R to T state, triggered by reductions in the PO_2_ and pH, occurs during blood flow within peripheral capillaries; and that single peripheral capillary analyses mirror events in capillary networks. These assumptions are discussed later in this manuscript. Equations used in the different calculations described below are provided in Appendix S1.

### 2.1 Calculation of the ↑ AC[COHb]

Calculations of the AC/[COHb] and the End‐AC[COHb] as the [COHb] increased were performed using normal constants measured in healthy adult men. The uptake of CO into alveolar capillary blood was calculated by multiplying the steady‐state DL_CO_ by the difference between mean alveolar P_CO_ and the BKP‐P_CO_, which produced uptake values with units of mL/min. Using the data described by Filley et al. (1954) that described the effects of CO breathing in young male resting subjects, our calculations used a DL_CO_ value of 25 mL/(min × mmHg), along with a mean alveolar P_CO_ equal to 60% of the inspired P_CO_. The BKP‐P_CO_ was calculated using a standard method that adjusts DL_CO_ measurements for back pressure (Sansores et al., 1992) as equal to (Inlet‐[COHb] × mean alveolar capillary PO_2_)/(M_R_ × mean alveolar capillary [O_2_Hb]). As described above, these calculations used a 100 mmHg mean alveolar capillary PO_2_. The [O_2_Hb] was calculated as 100% minus the Inlet‐[COHb]. The calculated CO uptake (mL/min) was then divided by a resting rate of blood flow in alveolar capillaries equal to a cardiac output, 5000 mL/min (Comroe et al., 1955), giving mL CO uptake into 100 mL blood. This unit was converted to COHb % sat. by dividing the mL CO uptake into 100 mL blood by 20.1 and multiplying the result by 100. The value 20.1 represents the CO capacity in mL/100 mL blood for a [Hb] of 15 g/100 mL blood.

### 2.2 Calculation of the end‐AC[COHb]

End‐AC[COHb] values were calculated as the sum of the Inlet‐[COHb] and the ↑ AC[COHb].

### 2.3 Calculation of the entry‐P_CO_

Following evidence described in the Introduction that Chem Eq of terms in the ReplR is achieved in blood entering peripheral capillaries, the Entry‐P_CO_ of peripheral capillaries was calculated as equal to ([Entry‐COHb] × Entry‐PO_2_)/(M_R_ × Entry‐[O_2_Hb]) (Coburn, 2018). The Entry‐PO_2_ was kept constant at 100 mmHg and the [O_2_Hb] determined for different [COHb] as described above. As discussed in the Introduction there would not be a significant change in the [COHb] as blood flowed from the lung to the Entry. Therefore, in calculations the Entry‐[COHb] was considered equal to the End‐AC[COHb]. For conditions where [COHb] were increased, [O_2_Hb] at a given PO_2_ were read from Roughton‐Darling plots shown in their Figure 1 (Roughton & Darling 1944).

### 2.4 Calculation of a mPC‐P_CO_

It is known that there are large variations in properties of different peripheral capillaries (Hudetz, 1997). But we only analyzed single capillaries. In one capillary, decreases from the Entry‐P_CO_ to the mPC‐P_CO_ were chosen to be 20% of the decrease that would be needed to occur if the mPC‐P_CO_ achieved Chem Eq with other terms of the ReplR. This capillary, which has a short blood flow transit time, is termed a 20% Capillary. The reason for this approach is that comparing 20% Capillary results with those obtained using a capillary that had a longer blood flow transit time that allowed Chem Eq to occur at the mPC‐P_CO_ site, termed a Chem Eq Capillary, made it possible to evaluate effects of different blood flow transit times on mPC‐P_CO_ values. These calculations are presented in the Section 4.

The following steps were performed to calculate the mPC‐P_CO_ of this 20% Capillary. This capillary was assigned a mean PO_2_ of 40 mmHg and that a R‐to‐T Hb transition had occurred. (i) A Chem Eq P_CO_ was calculated for a given [COHb] and a Hb T state using an [O_2_Hb] corresponding to the 40 mmHg PO_2_ justified by the 3–5 msec T_1/2_ of the dissociation of O_2_Hb (Holland, 1969b) (ii) This difference was multiplied by 0.2 to simulate the P_CO_ decrease in blood flowing from the Entry of the 20% Capillary to its mPC‐P_CO_. (iii) This value was subtracted from the Entry‐P_CO_ giving the mPC‐P_CO_. In these calculations the mean capillary [COHb] used in mPC‐P_CO_ calculations was considered to be the same as the Entry‐[COHb]. As with the argument given above that the Entry‐[COHb] is nearly equal to the End‐AC[COHb], the small decreases in the mPC‐P_CO_ from the Entry‐P_CO_, calculated later in this article, cannot evoke a significant change in the [COHb]. The mPC‐P_CO_ of Chem‐eq Capillaries was calculated as equal to the Chem Eq P_CO_ determined as described above. Note multiple examples of mPC‐P_CO_ calculations are shown in the Appendix S1.

### 2.5 Determining a mTiss‐P_CO_

mTiss‐P_CO_ were defined as extracellular tissue fed by the single capillary analyzed in this study. Values of mTiss‐P_CO_ were assumed to be equal to the mPC‐P_CO_ of 20% Capillaries that, as described above, had a mPC‐PO_2_ of 40 mmHg and where Hb was in a T state.

### 2.6 Calculation of the Inlet‐P_CO_

The values of P_CO_ in blood entering alveolar capillaries (Inlet‐P_CO_) were not used in any calculation performed in this study but are used in a figure included later in this article that shows P_CO_ differences at different cycle sites. The Inlet‐P_CO_ was calculated under the assumption that Chem Eq of the ReplR is established as blood flows from peripheral tissues to alveolar capillaries, and that Hb is in the T state upon entering these alveolar capillaries. When the Inlet‐[COHb] was 1% sat. an Inlet‐PO_2_ of 40 mmHg (equal to a normal mixed venous PO_2_ (Comroe et al., 1955)), was used in calculations. When the [COHb] was elevated, Inlet‐PO_2_ values were determined as inversely proportional to the Inlet‐[COHb]. Inlet‐[O_2_Hb] values were determined as described above. M_T_ values of 123 were used in these calculations.

## 3 RESULTS

**FIGURE 2:**
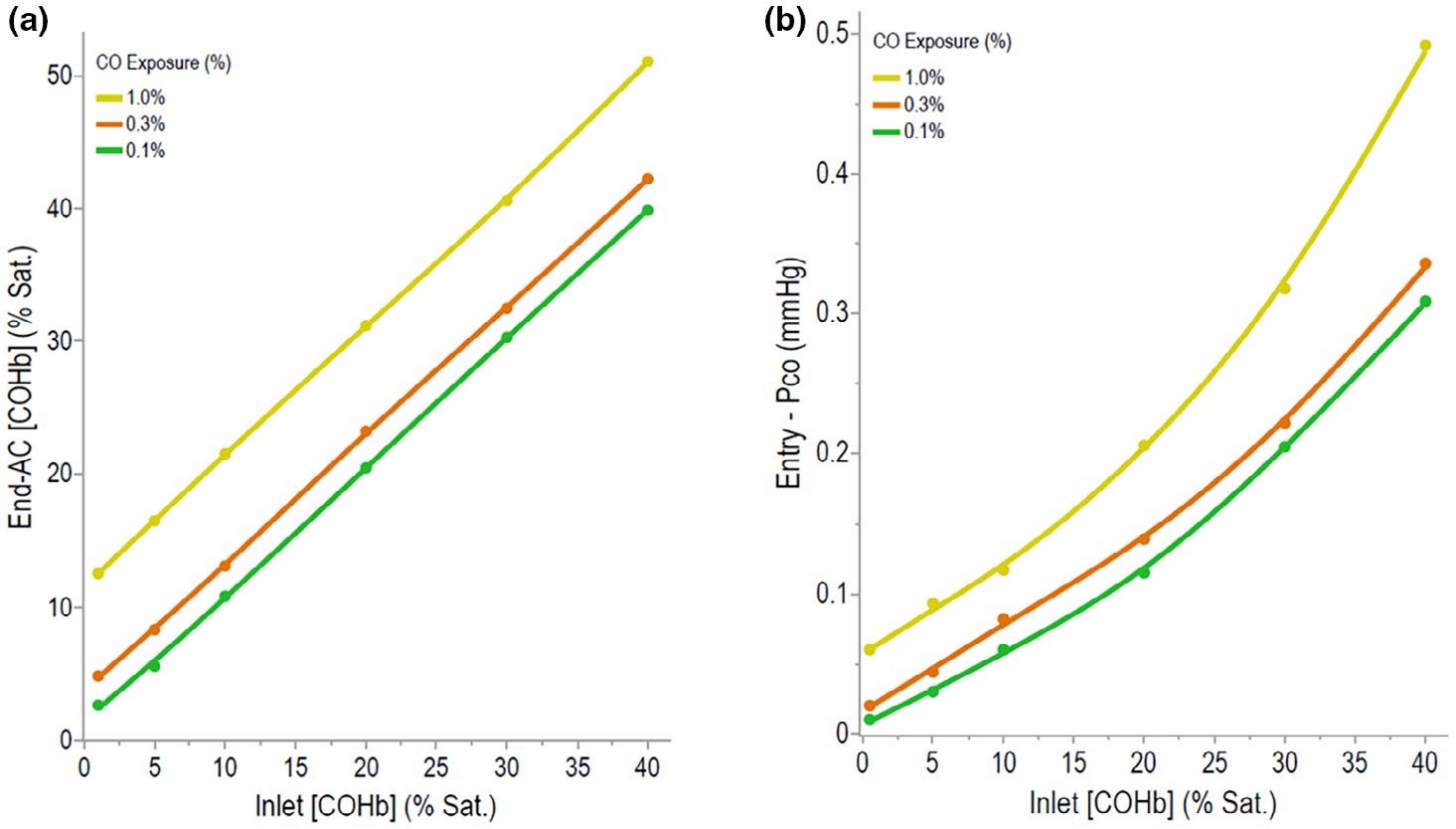
Effects of increases in the Inlet‐[COHb] due to breathing 0.1%, 0.3%, or 1% CO. on the (a) End‐AC[COHb]) and the (b) Entry‐P_CO_. Data were taken from Table 1.

**FIGURE 3:**
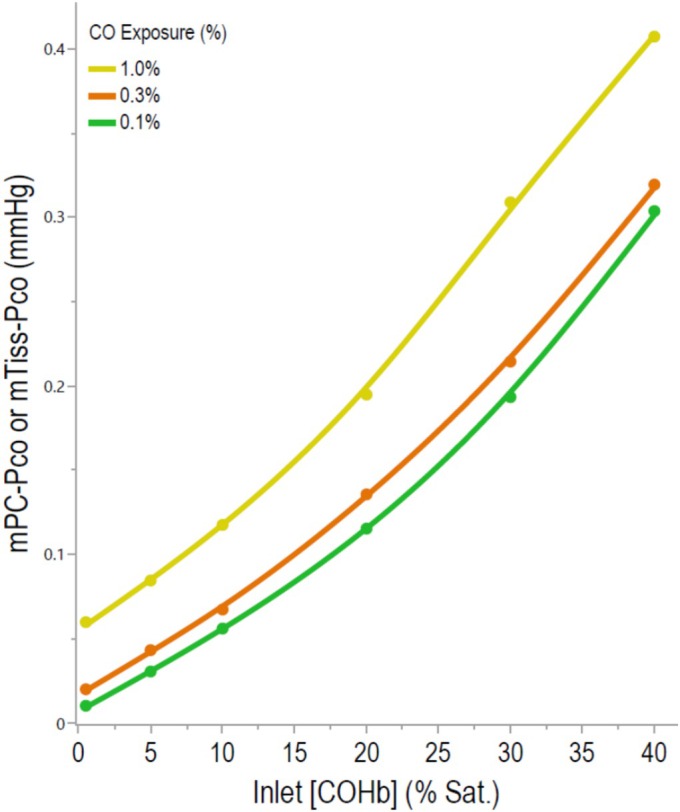
Increases in a mPC‐ P_CO_ and mTiss‐P_CO_ of a tissue during constant breathing of 0.1%, 0.3%, or 1.0% CO as the Inlet‐[COHb] increased. The mTiss‐P_CO_ values were considered to be in equilibrium with the mPC‐P_CO_ of 20% capillaries that had a mPC‐PO_2_ of 40 mmHg and where Hb had transitioned from an R to a T state. Differences in the plots shown in this figure compared to plots shown in Figure 2b are a result of P_CO_ decreases occurring in blood flowing within these capillaries. Data were taken from Table 1.

**FIGURE 4:**
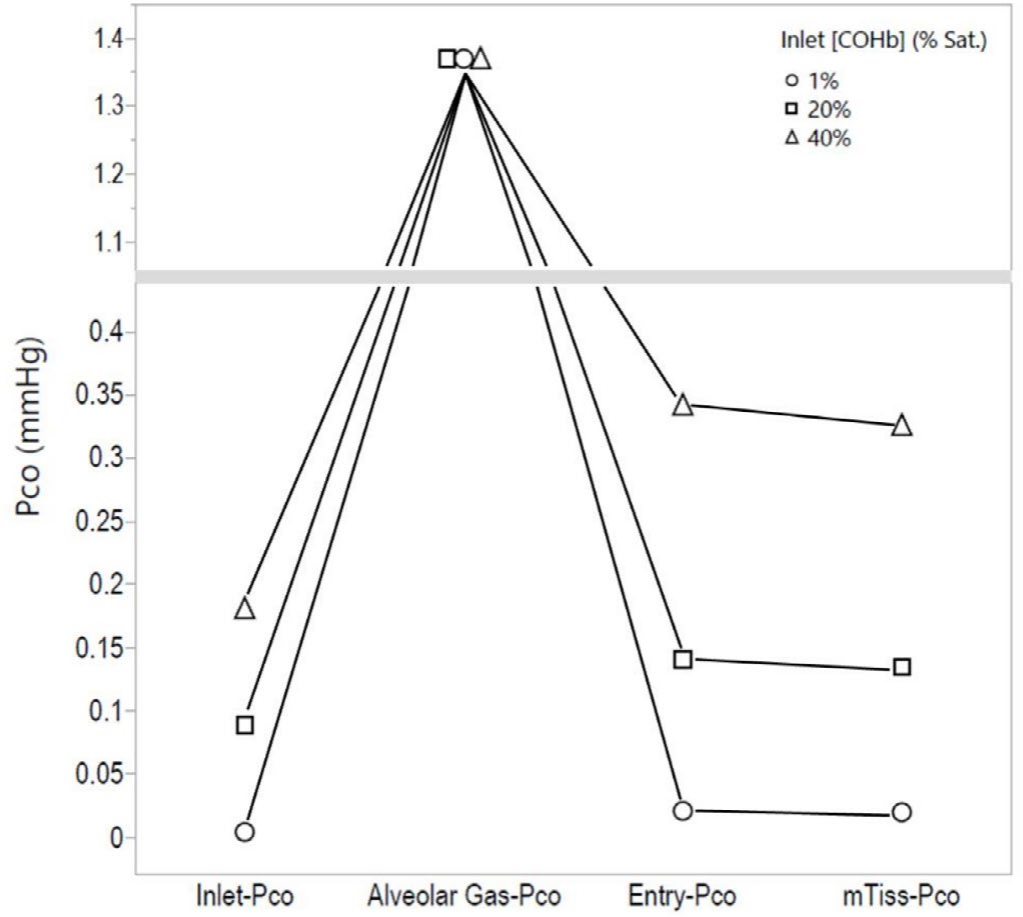
Single cycle changes in the P_CO_ at different cycle sites driven by a continuous inhalation of 0.3% CO: Single cycle data obtained at Inlet‐[COHb] of 1%, 20%, and 40%. The end‐alveolar P_CO_ could not be calculated. The mTiss‐P_CO_ is assumed equal to the mPC‐P_CO_ not shown in this figure. Data were taken from Table 1.

Table 1 lists the calculated increases of [COHb] or P_CO_ occurring at different CO cycle sites during the constant breathing of 0.1%, 0.3%, or 1.0% CO as the Inlet‐[COHb] increased from 1 to 5%, 10%, 20%, 30%, or 40% sat. Single cycle data at each Inlet‐[COHb] (see horizontal data in this table). Figure 2a,b show plots of End‐AC[COHb] and Entry‐P_CO_ increases as the Inlet‐[COHb] increased during the breathing of the different [CO]. Figure 3 shows plots of increases in a mTiss‐P_CO_ or mPC‐P_CO_ as a function of the Inlet‐[COHb] and the breathed [CO]. The plots shown in Figure 4 describe changes in the P_CO_ at different cycle sites driven by 0.3% CO breathing.

**TABLE 1:** Effects of 0.1%, 0.3%, and 1.0% CO breathing on CO cycle components.

Inlet [COHb] (% sat.)	Inlet Pco (mmHg)	Alveolar Pco—BKP‐Pco	↑AC [COHb] (% sat.)	End‐AC [COHb] (% sat.)	Entry‐Pco (mmHg)	mPC‐Pco and mTiss‐Pco (mmHg)^a^
0.1% CO breathing when the mean alveolar P_CO_ was 0.456 mmHg						
1.0	0.0043	0.454	1.14	2.14	0.010	0.010
5.0	0.022	0.432	1.08	6.08	0.030	0.030
10.0	0.041	0.405	1.00	11.00	0.056	0.055
20.0	0.089	0.341	0.856	20.86	0.119	0.118
30.0	0.140	0.259	0.650	30.65	0.203	0.196
40.0	0.181	0.146	0.365	40.37	0.314	0.301
0.3% CO breathing when the mean alveolar P_CO_ was 1.37 mmHg						
1.0	0.0043	1.37	3.43	4.43	0.021	0.020
5.0	0.022	1.35	3.38	8.38	0.042	0.041
10.0	0.041	1.32	3.30	13.30	0.070	0.069
20.0	0.089	1.26	3.15	23.15	0.141	0.135
30.0	0.140	1.17	2.93	32.93	0.220	0.212
40.0	0.181	1.07	2.68	42.68	0.342	0.326
1.0% CO breathing when the mean alveolar P_CO_ was 4.56 mmHg						
1.0	0.0043	4.56	11.40	12.40	0.065	0.054
5.0	0.022	4.54	11.35	16.35	0.090	0.087
10.0	0.041	4.51	11.30	21.30	0.123	0.120
20.0	0.089	4.47	11.18	31.18	0.205	0.199
30.0	0.140	4.37	10.95	40.95	0.321	0.308
40.0	0.181	4.26	10.65	50.65	0.480	0.406

*Note*: Horizonal data show single cycle values at a given Input‐[COHb]. When Inlet‐[COHb] were 1%, 5%, 10%, 20%, 30%, or 40% sat., BKP‐P_CO_ were 0.002, 0.024, 0.051, 0.110, 0.197 and 0.310 mmHg, respectively.

^a^ These data are results of calculations that used a single 20% capillary that had a mPC‐PO_2_ of 40 mmHg and where Hb had transitioned from an R to a T state.

## 4 DISCUSSION

### 4.1 Justifications of assumptions made in these calculations

The assumption that the alveolar PO_2_ does not change as the [COHb] increases was analyzed using the alveolar air equation (Alveolar PO_2_ = [P_ATM_ – P_H2O_] x Fi_O2_ – Pa_CO2_/RQ), where the RQ is the ratio of O_2_ uptake to CO_2_ excretion rates, so the issue is whether or not the arterial partial pressure of CO_2_ (Pa_CO2_) and RQ change as the [COHb] increases. Measurements of the Pa_CO2_ in CO poisoned humans show it varied in different patients, but many had normal values (Benaissa et al., 2003; Lebby et al., 1989; Vogel & Gleser, 1972). Considering the RQ, it has been reported that O_2_ consumption rates do not change in CO poisoned humans over the range of [COHb] increases considered in the present study (Smithline et al., 2003), but CO_2_ excretion rates were not measured in this study. If the assumption is not valid and the arterial PO_2_ decreases as the [COHb] increases, the ↑AC[COHb] and downstream P_CO_ would be larger than given in Table 1 and Figures 3 and 4. However, a goal of the study, to describe physiological variables involved in determining mTiss‐P_CO_ values, would not be influenced.

The assumption that the ReplR becomes activated due to decreasing PO_2_ levels that occur as blood flows within peripheral capillaries: We chose to discuss this assumption using data obtained in studies of the cerebral circulation (Hudetz, 1997). In this circulation, there are large variations in blood flow transit times in different capillaries of 0.5‐to‐1.8‐micron lengths, which ranged from 100 to 300 msec. A 300 msec transit time would be equivalent to 7 T_1/2_ of the ReplR. This suggests that this assumption is valid, but probably only for long capillaries.

The assumption that transition of Hb from the R‐to‐T state is rapid enough to be triggered by reductions in the PO_2_ and pH that occur during blood flow within peripheral capillaries: This is justified based on evidence that this transition occurs in 1 to 20 microsecs (Ahmed et al., 2020) and can be considered to parallel decreases in the PO_2_. Further evidence is that this transition is associated with two fast physiological processes that take place in blood flowing through peripheral tissues: (i) the dissociation of oxygen from O_2_Hb (Holland, 1969b), and (ii) the Bohr shift, which is driven by increases in P_CO2_ and corresponding decreases in pH (Ahmed et al., 2020; Forster & Steen, 1968). Thus the assumption that R‐to‐T state transitions are a determinant of the mPC‐P_CO_ and mTiss‐P_CO_ seems strongly supported.

The assumption there is a rapid equilibration of CO between blood and tissue was discussed in the Introduction: It depends on what is meant by “rapid”. Note, as cited in the Introduction, in anesthetized dog experiments there was no lag in the time required to mix in extravascular stores after administration of CO compared to mixing of injected erythrocytes that had elevated COHb and other evidence given in the Section 1.

The assumption that single peripheral capillary data obtained in this study mirrors events in capillary networks: This assumption is not supported.

### 4.2 Events at the different sites in the CO cycle

#### 4.2.1 At the alveolar site

Increases in the [COHb] which evoked BKP‐P_CO_ increases resulted in a decreased P_CO_ gradient between alveolar gas and blood in alveolar capillaries, ultimately resulting in a reduction in rates of pulmonary CO uptake. Results of calculations show that this effect is markedly different with different breathing [CO]. During 0.1% CO breathing, as the Inlet‐[COHb] increased from 1% to 40% sat. this P_CO_ gradient decreased from 0.454 to 0.146 mmHg; during 0.3% CO breathing—from 1.37 to 1.07 mmHg; and during 1.0% CO breathing—from 4.56 to 4.26 mmHg. Due to this reduced P_CO_ gradient as the Inlet‐[COHb] increased from 1% to 40% sat., the ↑AC[COHb] declined from 1.14% to 0.37% sat. during 0.1% CO breathing. However, during 1.0% CO breathing, the decrease in ↑AC[COHb] over the same Inlet‐[COHb] increase (1 to 40%) resulted in a reduction from 11.40 to only 10.65% sat. These results indicate that the relationships between these parameters vary depending on the inhaled [CO] and the change in the Inlet‐[COHb]. As a result, the relative influence of inhaled [CO] and Inlet‐[COHb] on both the End‐AC[COHb] and the P_CO_ at down‐stream sites in the CO cycle is altered. Since pulmonary CO uptake drives this CO cycle, these interactions exert control of CO cycling. These results imply that in humans breathing a low [CO], after a high Inlet‐[COHb] is achieved it is the history rather than the inhaled [CO] that mainly determines the cycling of CO to peripheral tissues. However, during higher [CO] breathing, illustrated in this article as 1.0% CO, alveolar CO uptake rates remained large even as the Inlet‐[COHb] increased. Possible clinical significance: an increased Inlet‐[COHb] in humans breathing low [CO] offers some protection from a further increase in toxicity by decreasing CO uptake, a protection which does not occur during high [CO] breathing.

#### 4.2.2 At the entry site

**FIGURE 5:**
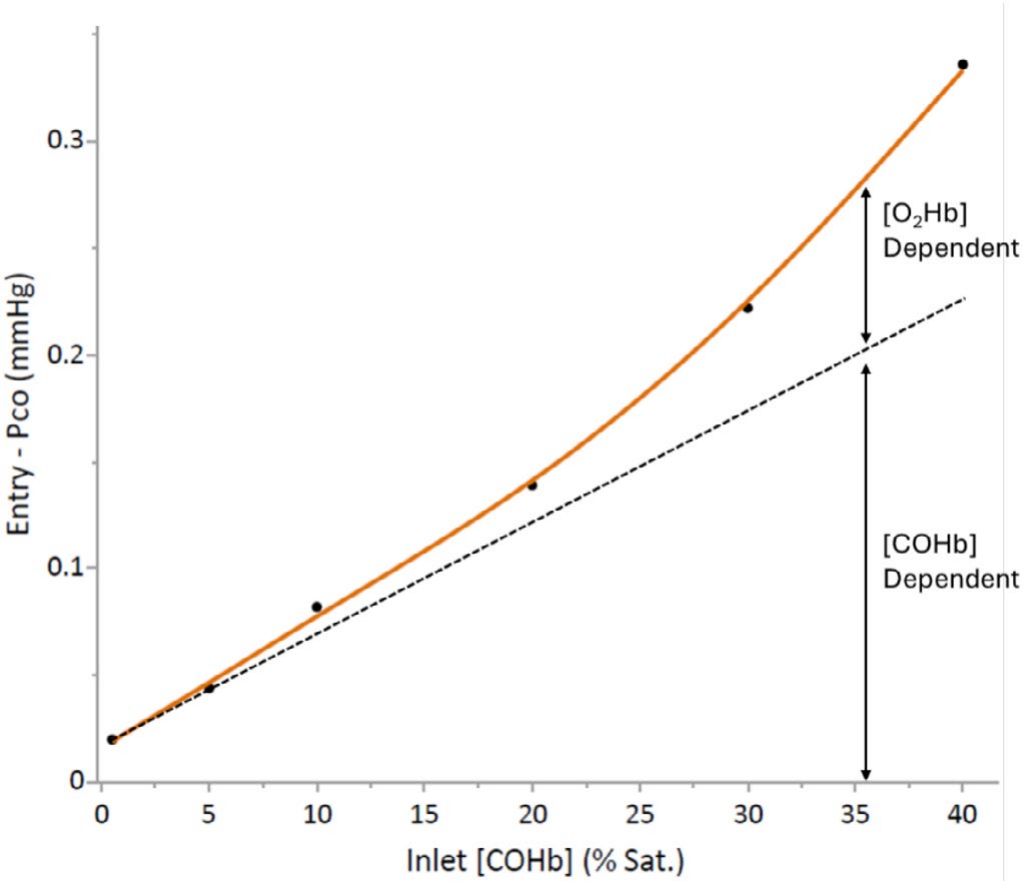
This figure illustrates the relative contributions of increases in the Entry‐[COHb] and decreases in the Entry‐[O_2_Hb] to increases in the Entry‐P_CO_ as the Inlet‐[COHb] increased. The 0.3% CO breathing data shown in Figure 2b were used in constructing this figure.

Evidence is given in the Introduction that the P_CO_ of blood at Entry sites is in a Chem Eq with other terms of the replacement reaction and, therefore, sensitive to the Entry‐[O_2_Hb] which decreases as [COHb] increases. The Entry‐PO_2_ is also a determinant of the Entry‐P_CO_; however, in these calculations this variable was kept constant at 100 mmHg. The plot shown in Figure 5 illustrates [COHb]‐dependent and [O_2_Hb]‐dependent increases of the Entry‐P_CO_. The red line plotted in this figure is taken from data shown in Figure 2a, whereas the dashed line was created as proportional to the [COHb]. Similar plots using 0.1 and 1.0% CO breathing data allowed a similar determination of the contribution of [O_2_Hb] decreases to Entry‐P_CO_ increases (data not shown). Results indicated that when the Inlet‐[COHb] was 20% sat., decreases in the Entry‐[O_2_Hb] evoked 16, 9, and 9% increases in the Entry‐P_CO_ during 0.1, 0.3, and 1.0% CO breathing, respectively. When the Inlet‐[COHb] was 40% sat., corresponding decreases in [O_2_Hb] resulted in the Entry‐P_CO_ increasing 39, 33 and 31% during 0.1, 0.3 and 1.0% CO breathing, respectively. Findings support the concept of a significant [O_2_Hb]‐sensitive amplification of the Entry‐P_CO_ in peripheral capillaries and that a regulatory mechanism operates at this site in the CO cycle, especially under conditions of an elevated [COHb]. Evidence that this [O_2_Hb]‐sensitive amplification of the Entry‐P_CO_ is transmitted downstream to the P_CO_ values in extravascular tissues is indicated by the upward curvature of the Inlet‐[COHb] vs. mPC‐P_CO_ or mTiss‐P_CO_ plot shown in Figure 3.

#### 4.2.3 At the mPC‐P_CO_ site

We studied different variables that could influence the mPC‐P_CO_ in different peripheral capillaries: the importance of a R to T state transition and effects of different mean capillary PO_2_ values and blood flow transit times.

#### 4.2.4 A transition from a Hb R to a T state

New calculations assessed the significance of this transition as a determinant of the mPC‐P_CO_ calculated using either the M_R_ = 218 or the M_T_ = 123. These calculations considered blood flowing within 20% capillaries and used 0.3% CO breathing data shown in Table 1, an Entry‐[COHb] of 23.15% sat., a mean capillary PO_2_ of 40 mmHg, and an [O_2_Hb] of 63.7% sat. determined as described in the Section 2. Results indicated that a transition from the R to the T state would evoke an increase in the mPC‐P_CO_ from 0.126 to 0.135 mmHg. When the Entry‐[COHb] was 42.68% sat. and using the same 0.3% CO breathing parameters and an [O_2_Hb] of 54% sat., this transition would evoke a mPC‐P_CO_ increase from 0.304 to 0.326 mmHg. Similar increases occurred in calculations using 0.1 and 1.0% CO breathing data. These differences provide evidence that the R‐to‐T transition of Hb is a determinant of a mPC‐P_CO_ and, therefore, a mTiss‐P_CO_. Although these effects are small, this transition needs to be considered as a factor in the physiology of CO uptake and poisoning.

Decreases in mean P_CO_ of blood traveling through peripheral capillaries, not increases, were shown by the differences between Entry‐P_CO_ and both mPC‐P_CO_ and mTiss‐P_CO_ values listed in Table 1 and plotted in Figure 4. The reason for this is that P_CO_ decreases resulting from operation of the ReplR are larger than increases resulting from R‐to‐T transitions. If R‐to‐T transitions occur slowly, mPC‐P_CO_ and mTiss‐P_CO_ values provided in this article would be overestimated. The transition of Hb from the T‐to‐R state as blood enters alveolar capillaries could also influence CO cycling by causing CO uptake to increase as a result of an increase in the affinity of CO binding to Hb in the R state. This mechanism has not been analyzed in this study.

#### 4.2.5 Effects of different mean capillary PO_2_ on the mPC‐P_CO_

Peripheral capillaries supplying different tissues, or even different regions within the same tissue, exhibit distinct mean PO_2_ values, but mPC‐P_CO_ data listed in Table 1 and plotted in Figure 3 were calculated for single capillaries which had mean PO_2_ of 40 mmHg. Here we have extended analyses to evaluate how sensitive the mPC‐P_CO_ is to changes in the mean capillary PO_2_. We first calculated mPC‐P_CO_ values for capillaries that fed tissues that had a low O_2_ uptake/blood flow rate and, therefore, a high mean PO_2_. Calculations were performed using mean PO_2_ levels of 50 and 60 mmHg. These calculations used 20% capillaries which were in the T allosteric state, and 0.3% CO breathing data listed in Table 1 and were performed as described in Section 2. Results indicated that when the Entry‐[COHb] was 23.15% sat. and the Entry‐P_CO_ 0.141 mmHg, using 50 and 60 mmHg mean PO_2_ in calculations resulted in changes in the mPC‐P_CO_ from the 40 mmHg value of 0.135 mmHg to 0.126 and 0.147 mmHg, respectively. When the Entry‐[COHb] was 42.68% sat. and the Entry‐P_CO_ 0.342 mmHg, using 50 and 60 mmHg mean PO_2_ in calculations changed the mPC‐P_CO_ from the 40 mmHg value of 0.326 mmHg to 0.276 and 0.351 mmHg, respectively. These differences in effects on the mPC‐P_CO_ of increases in the mean PO_2_ from 40 to 50 and from 40 to 60 mmHg shown above are due to changes in the shape of Hb O_2_ dissociation curve that occurs at these high [COHb]. Some of these calculations are shown in Appendix S1.

Decreases in mean capillary‐PO_2_ as [COHb] increases occur due to a COHb‐evoked leftward shift (higher O_2_ affinity) in the O_2_Hb dissociation curve (Roughton & Darling, 1944). These PO_2_ decreases could cause compensatory vasodilation and increased blood flow affected via metabolism‐blood flow coupling mechanisms (also termed O_2_ uptake‐blood flow coupling mechanisms) (reviewed by Coburn (2020)); however, it is uncertain if this occurs during different severities of CO poisoning because there is evidence that metabolism‐blood flow coupling is disabled under this condition (Coburn, 2020). Increased blood flow to a tissue, if it does not entirely involve recruited capillaries, could decrease capillary blood flow transit times evoking an effect on the mPC‐P_CO_ as discussed in the next section of this article. Inhibition of carotid body O_2_ sensors during CO poisoning (Lahiri et al., 1981) could result in changes in the blood flow and the mean PO_2_ in some peripheral vascular beds. Calculations were performed to assess the effects of decreases in this PO_2_ on the mPC‐P_CO_. Using 20% Capillaries as the model again: during 0.3% CO breathing, when the Entry‐[COHb] was 23.15% sat. and the Entry‐P_CO_ was 0.141 mmHg, reducing the mean PO_2_ from 40 mmHg to 30 or 20 mmHg had little effect on the calculated mPC‐P_CO_, reducing to 0.133 and 0.132 mmHg, respectively. Likewise when the Entry‐[COHb] was 42.68% sat. and the Entry‐P_CO_ 0.342 mmHg, decreasing the mean PO_2_ from 40 to 30 or 20 mmHg only reduced the mPC‐P_CO_ from the 40 mmHg value of 0.326 mmHg to 0.313 and 0.310 mmHg, respectively. Examples of these calculations are shown in Appendix S1. Similar findings were obtained using 0.1 and 1.0% CO breathing data. Although the ReplR driven by PO_2_ decreases in blood flowing from the Entry value of 100 mmHg to a mPC‐P_O2_ of 40 mmHg evokes P_CO_ decreases, no further decrease occurs as the mean capillary PO_2_ decreased below this value. The explanation for this requires further analysis but likely is due to the shape of O_2_ dissociation curves plotted for different [COHb] (Roughton & Darling, 1944) where PO_2_ decreases below 40 mmHg occur nearly in parallel with decreases in the [O_2_Hb].

#### 4.2.6 Effects of blood flow transit times on the mPC‐P_CO_

As described above, capillaries which have longer blood flow transit times than occur in 20% Capillaries would have larger decreases in their P_CO_ and mPC‐P_CO_ than those shown in Table 1. But how large are these differences? We calculated mPC‐P_CO_ values in both a 20% Capillary and a Chem‐Eq Capillary using 0.3% CO breathing data listed in this table, and where both capillary types had the same Entry‐P_CO_ and Entry‐[COHb], a mean capillary PO_2_ of 40 mmHg and were in the T Hb allosteric state. Results are listed in Table 2.

**TABLE 2:** Effects of increasing capillary blood flow transit times on the mPC‐P_CO_.

Entry‐[COHb] (% sat.)	Entry‐Pco (mmHg)	20% capillary mPC‐Pco (mmHg)	Chem‐Eq capillary mPC‐Pco (mmHg)
13.30	0.070	0.069	0.064
23.15	0.141	0.135	0.118
42.68	0.342	0.326	0.262

*Note*: The 20% Capillary had a “short” blood flow transit time and the Chem‐Eq Capillary a “long” blood flow transit time.

These results show that a larger decrease from the Entry‐P_CO_ to the mPC‐P_CO_ occurred in the Chem‐Eq Capillary than in the 20% Capillary and that differences were larger at high Entry‐[COHb] levels. These data provide the first estimate of the importance of peripheral blood flow transit times in determining P_CO_ decreases in blood flowing in these capillaries and the mPC‐P_CO_.

### 4.3 Limitations of the study

(i). The study considered only a condition where the arterial PO_2_ was 100 mmHg. There would be large effects on the P_CO_ and [COHb] of cycle components in humans suffering from CO poisoning scenarios that resulted in arterial hypoxemia. (ii) Effects of metabolic acidosis were not considered. (iii) There is evidence cited in the Introduction that the minute ventilation and cardiac output do not increase as the [COHb] increases up to 30 to 35% sat. However, it is not. certain if this applies to higher [COHb] levels. It is possible that 40% sat. [COHb] data reported in the present article has errors if these parameters increased. (iv) Calculations used normal constants relevant to adult healthy men and did not include other life history states in humans. (v) We considered only single peripheral capillaries; (vi) The comparison of the physiology of the 20% Capillary and Chem Eq Capillary could not define them with information about their length and blood flow transit times. (vii) Many of the calculations were only performed using a 20% Capillary; (viii) It is uncertain if results obtained using the single capillary approach can be accurately extrapolated to capillary networks. (ix) An understanding of factors that influence the mTiss‐P_CO_ of different tissues under different conditions is incomplete: this is a frontier issue and we are only at the stage of listing factors.

## 5 CONCLUSIONS

Analyzing CO uptake as a cycle provides a new approach to the understanding of the physiology of CO uptake and poisoning. In this study we considered conditions where different inspired [CO] were constantly breathed, and analyzed physiological events that occur at different sites in this cycle: the ↑ AC[COHb], the End‐AC[COHb], the Entry‐P_CO_, the mPC‐P_CO_, and the Inlet‐[COHb]. These analyses emphasized the central role of the ReplR in the physiology of acute CO uptake. The operation of the CO cycle is governed by interactions between the mean alveolar P_CO_, the BKP‐P_CO_, and the Inlet‐[COHb], with these relationships shifting as the Inlet‐[COHb] increases. Decreases in the arterial [O_2_Hb] due to [COHb] increases result in amplification of the Entry‐P_CO_ and contribute to regulation of the cycle. Additionally, cycling between hemoglobin R and T allosteric states influences the dynamics of CO uptake. Within a peripheral capillary, its mPC‐P_CO_ is determined by several interacting factors, including the Entry‐[COHb] and ‐P_CO_, the mean capillary PO_2_, the capillary blood flow transit time, the kinetics of the ReplR, and hemoglobin allosteric state transitions. These findings indicate that multiple components of the CO cycle can regulate or modulate CO uptake and influence both the mPC‐P_CO_ and the mTiss‐P_CO_.

## AUTHOR CONTRIBUTIONS

**Ronald F. Coburn:** Conceptualization; data curation; formal analysis; funding acquisition; investigation; methodology; project administration; resources; software; supervision; validation; visualization. **Michael S. Tift:** Funding acquisition; resources; software; supervision; validation; visualization.

## FUNDING INFORMATION

MST was supported by NSF Award Numbers 1927616 and 2042043.

## CONFLICT OF INTEREST STATEMENT

All authors declare no conflicts of interest.

## ETHICS STATEMENT

Appropriate animal and human use approvals were in place at the time of the experiments.

## Supporting information


Appendix S1.

## Data Availability

The data that supports the findings of this study are available in Tables in text and are also available from the corresponding author (MST), upon reasonable request.

## References

[phy270858-bib-0001] Abboud, R. J. , Andersson, G. , & Coburn, R. F. (1974). Evaluation of stagnant pulmonary capillary blood during breath holding in dogs. Journal of Applied Physiology, 37, 397–409. 4413318 10.1152/jappl.1974.37.3.397

[phy270858-bib-0002] Ahmed, M. H. , Ghatge, M. S. , & Safo, M. K. (2020). Hemoglobin: Structure, function, allostery. Sub‐Cellular Biochemistry, 94, 345–382. 10.1007/978-3-030-41769-7_14 32189307 PMC7370311

[phy270858-bib-0003] Asmussen, E. , & Chiodi, H. (1941). The effect of hypoxemia on ventilation and circulation in man. The American Journal of Physiology, 132, 426–436.

[phy270858-bib-0004] Ayres, S. M. , Giannelli, S., Jr. , & Mueller, H. (1970). Myocardial and systemic responses to carboxyhemoglobin. In Biological Effects of Carbon Monoxide (Vol. 174, pp. 268–293). Annals of the New York Academy of Sciences. 10.1111/j.1749-6632.1970.tb49795.x 5289605

[phy270858-bib-0005] Benaissa, M. L. , Megarbane, B. , Borron, S. W. , & Baud, F. J. (2003). Is elevated plasma lactate a useful marker in the evaluation of pure carbon monoxide poisoning. Intensive Care Medicine, 29(6), 1372–1375. 12856122 10.1007/s00134-003-1866-0

[phy270858-bib-0006] Bruce, E. N. , Bruce, M. C. , & Erupaka, K. (2008). Prediction of the rate of uptake of carbon monoxide from blood by extravascular tissues. Respiratory Physiology & Neurobiology, 161, 142–159. 18313993 10.1016/j.resp.2008.01.004 PMC2430150

[phy270858-bib-0007] Chakraborty, S. , Balakotaiah, V. , & Bidani, A. (2004). Diffusing capacity reexamined: Relative role of diffusion and chemical reaction of O_2_, CO, CO_2_ and NO. Journal of Applied Physiology, 97, 2284–2302. 10.1152/japplphysiol.00469.2004 15322062

[phy270858-bib-0008] Chiodi, H. , Dill, D. B. , Consolazio, F. , & Horvath, S. H. (1941). Respiratory and circulatory responses to acute carbon monoxide poisoning. The American Journal of Physiology, 134, 683–692. 10.1152/ajplegacy.1941.134.4.683

[phy270858-bib-0009] Coburn, R. F. (2018). The partial pressure of carbon monoxide in human tissues calculated using a parallel capillary‐tissue cylinder model. Journal of Applied Physiology, 124, 761–768. 10.1152/japplphysiol.00833 29357489

[phy270858-bib-0010] Coburn, R. F. (2020). Coronary and cerebral metabolism‐blood flow‐coupling and pulmonary alveolar ventilation‐blood flow coupling may be disabled during acute carbon monoxide poisoning. Journal of Applied Physiology, 129, 1039–1050. 10.1152/japplphysiol.00172.2020152/jappl.1981.50.3.580 32853110

[phy270858-bib-0011] Coburn, R. F. (2021). Effects of increases in carboxyhemoglobin % saturation and tissue hypoxia on carbon monoxide binding in canine skeletal and heart muscle extravascular tissue. Journal of Applied Physiology, 131, 64–71. 10.1152/japplphysiol.00031.2021 34013749

[phy270858-bib-0012] Coburn, R. F. (2022). Carbon monoxide (CO), nitric oxide and hydrogen sulfide signaling during acute CO poisoning. Frontiers in Pharmacology, 12, 83024. 10.3389/fphar.2021.830241 PMC8972574 35370627

[phy270858-bib-0013] Coburn, R. F. , Forster, R. E. , & Kane, P. B. (1965). Considerations of the physiological variables that determine the blood carboxyhemoglobin concentration in man. The Journal of Clinical Investigation, 44, 1899–1910. 5845666 10.1172/JCI105296 PMC289689

[phy270858-bib-0014] Collman, J. P. , Brauman, J. I. , Halbert, T. R. , & Suslick, K. S. (1976). Nature of O_2_ and CO binding to metalloporphyrins and heme proteins. Proceedings of the National Academy of Sciences of the United States of America, 73, 3333–3337. 1068445 10.1073/pnas.73.10.3333 PMC431107

[phy270858-bib-0015] Comroe, J. H. , Forster, R. E. , DuBois, A. B. , Briscoe, W. A. , & Carlsen, E. (1955). The Lung. Year Book Publishers, Inc.

[phy270858-bib-0016] Di Cera, E. , Doyle, M. L. , Connelly, P. R. , & Gill, S. L. (1987). Carbon monoxide binding to human hemoglobin Ao. The Biochemist, 26, 6494–6502. 10.1021/bi00394a031 3427020

[phy270858-bib-0017] Filley, G. F. , MacIntosh, D. J. , & Wright, G. W. (1954). Carbon monoxide uptake and pulmonary diffusing capacity in normal subjects at rest and during exercise. The Journal of Clinical Investigation, 33, 530–539. 10.1172/JCI102923 13152192 PMC1087267

[phy270858-bib-0018] Forster, R. E. (1964). Diffusion of gases. In W. O. Fenn & H. Rahn (Eds.), Handbook of Physiology, Respiration (Vol. 1, pp. 839–872). American Physiological Society, Section 3.

[phy270858-bib-0019] Forster, R. E. , & Steen, J. B. (1968). Rate limiting processes in the Bohr shift in human red cells. The Journal of Physiology, 196, 541–562. 5664232 10.1113/jphysiol.1968.sp008522 PMC1351763

[phy270858-bib-0020] Greiner, T. H. (1997). Carbon monoxide concentration table. Table Pub # AEN‐172, Department of Agriculture and Bioengineering, Iowa State University.

[phy270858-bib-0021] Haldane, J. , & Smith, J. L. (1896). The oxygen tension of arterial blood. The Journal of Physiology (London), 20, 497–520. 10.1113/jphysiol.1896.sp000634 PMC1512613 16992350

[phy270858-bib-0023] Holland, R. A. B. (1969a). Rate at which carbon monoxide replaces O_2_ from O_2_Hb in red cells of different species. Respiration Physiology, 7, 43–63. 10.1016/0034.5687(69)90068-1 5809094

[phy270858-bib-0024] Holland, R. A. B. (1969b). Rate of O_2_ dissociation from O_2_Hb and relative combination rate of CO and O_2_ in mammals at 37°C. Respiration Physiology, 7, 30–42. 10.1016/0034-5687(69)90067-X 5817714

[phy270858-bib-0025] Holland, R. A. B. (1970). Reaction rates of carbon monoxide and hemoglobin. In Biological Effects of Carbon Monoxide (Vol. 174, pp. 154–171). Annals of the New York Academy of Sciences. 10.1111/j.1749-6632.1970.tb49782.x 4943971

[phy270858-bib-0026] Hudetz, A. G. (1997). Blood flowing in the cerebral capillary network: A review emphasizing observations with intravital microscopy. Microcirculation, 4, 233–252. 10.3109/107396890019467870 9219216

[phy270858-bib-0027] Hughes, J. M. B. , & Bates, D. V. (2003). Historical review: The carbon monoxide diffusing capacity (DLCO) and its membrane (Dm) and red cell components. Respiratory Physiology & Neurobiology, 138, 115–142. 14609505 10.1016/j.resp.2003.08.004

[phy270858-bib-0028] Lahiri, S. , Mulligan, E. , Nishino, T. , Mokashi, A. , & Davies, R. O. (1981). Relative responses of aortic body and carotid body chemoreceptors to carboxyhemoglobin. Journal of Applied Physiology, 50, 580–586. 7251448 10.1152/jappl.1981.50.3.580

[phy270858-bib-0029] Lebby, T. I. , Zalenski, R. , Hryhorczuk, D. O. , & Leikin, J. B. (1989). The usefulness of the arterial blood gas in pure carbon monoxide poisoning. Veterinary and Human Toxicology, 31, 138–149. 2929122

[phy270858-bib-0030] Luomanmaki, K. , & Coburn, R. F. (1969). Effects of metabolism and distribution of carbon monoxide on blood and body stores. The American Journal of Physiology, 217, 354–363. 5799358 10.1152/ajplegacy.1969.217.2.354

[phy270858-bib-0031] Penney, D. G. (1988). A review: Hemodynamic response to carbon monoxide. Environmental Health Perspectives, 77, 121–130. 3289904 10.1289/ehp.8877121 PMC1474537

[phy270858-bib-0032] Peterson, J. E. , & Stewart, R. D. (1970). Absorption and elimination of carbon monoxide in inactive young men. Archives of Environmental Health: An International Journal, 21, 165–170. 10.1080/00039896.1970.10667215 5430002

[phy270858-bib-0033] Power, G. G. (1968). Solubility of O_2_ and CO in blood and pulmonary and placental tissues. Journal of Applied Physiology, 24, 468–472. 5643394 10.1152/jappl.1968.24.4.468

[phy270858-bib-0034] Rodkey, F. L. , O'Neal, J. D. , & Collison, H. A. (1969). Oxygen and carbon monoxide equilibria of human adult hemoglobin at atmosphere and elevated pressure. Blood, 33, 57–65. 10.1152/ajplegacy.1944.141.1.17 5763633

[phy270858-bib-0035] Roughton, F. J. W. (1964). Transport of oxygen and carbon dioxide. In W. O. Fenn & H. Rahn (Eds.), Handbook of Physiology – Respiration (Vol. 1, pp. 767–825). American Physiological Society, Section 3.

[phy270858-bib-0036] Roughton, F. J. W. , & Darling, R. C. (1944). The effect of carbon monoxide on the oxyhemoglobin dissociation curve. The American Journal of Physiology, 141, 17–31.

[phy270858-bib-0037] Roughton, F. J. W. , & Forster, R. E. (1957). Relative importance of diffusion and chemical reaction rates in determining rate of exchange of gases in the human lung, with special reference to true diffusing capacity of pulmonary membranes and volume of blood in lung capillaries. Journal of Applied Physiology, 11, 290–301. 13475180 10.1152/jappl.1957.11.2.290

[phy270858-bib-0038] Sansores, R. H. , Pare, P. , & Abboud, R. T. (1992). Effect of smoking cessation on pulmonary carbon monoxide diffusing capacity and capillary blood volume. American Journal of Respiratory and Critical Care Medicine, 146, 959–964. 10.1164/ajrccm/146.4.959 1416425

[phy270858-bib-0040] Smithline, H. A. , Ward, K. R. , Chilli, D. A. , Blake, H. C. , & Ricers, E. R. (2003). Whole body oxygen consumption and critical oxygen delivery and response to prolonged severe carbon monoxide poisoning. Resuscitation, 56, 97–104. 10.1016/s0300-9572(02)00272-1 12505745

[phy270858-bib-0041] Vogel, J. A. , & Gleser, M. A. (1972). Effect of carbon monoxide and oxygen transport during exercise. Journal of Applied Physiology, 32, 234–239. 4550276 10.1152/jappl.1972.32.2.234

