# Supplementary material for: A carbon monoxide cycle drives carbon monoxide uptake and poisoning
Source: Physiol Rep. 2026 Apr 15;14(8):e70858. doi: 10.14814/phy2.70858 (PMC13083032; doi:10.14814/phy2.70858)
Supplement: Supplementary file 1 — Appendix S1. [file PHY2-14-e70858-s001.docx]

Supplemental File

***Example calculations.*** These calculations all used 0.3% CO breathing data.

**Calculation:** **the Inlet-P_CO_:** using an Inlet-PO_2_ of 30 mmHg, an Inlet-[O2Hb] of 55.0% sat., a

MT of 123 and an Inlet-[COHb] of 20% sat..

**Inle**t- **P_CO_ = ([COHb] x PO_2_)/(MT x [O_2_Hb])**

Inlet-P_CO_ = (20.0 x 30.0)/(123 x 55.0) = 0.089 mmHg

**Calculation: the mean BKP-P_CO_:** using a mean AC [O_2_Hb] of 78.0% sat., a mean

AC-PO_2_ of 100 mmHg, a MR of 218, and an Inlet-[COHb] of 20% sat..

**BKP-P_CO_ = (Inlet-[COHb] x mean AC PO_2_)/(MR x mean AC [O_2_Hb])**

BKP-P_CO_ = (20 x 100)/(218 x 78) = 0.11 mmHg

**Calculations:** **the ↑ AC[COHb]:** using a DLCO of 25 ml/(min x mm Hg), an (alveolar Pco

1.37 mmHg - the mean BKP-Pco of 0.11 mmHg) = to 1.26 mmHg, an alveolar capillary

blood flow of 5000 ml/min, a blood CO capacity of 20.1 ml/100 ml (corresponding to a [Hb] of 15 grams/100 ml blood) and an Inlet-[COHb] of 20% sat..

**(i). CO uptake into AC blood = DLCO x (alveolar Pco - the mean BKP-Pco)**

CO uptake into AC blood = 25 x 1.26 = 31.5 ml/min

**(ii). CO uptake into AC blood given as ml/100 ml blood flow:** The CO uptake in

ml/min is divided by the ml/min alveolar capillary blood flow

↑ CO uptake into AC blood = 31.5/5000 = 0.63 ml per 100 ml blood flow

**(iii). ↑ AC[COHb] due to CO uptake:** ml CO uptake per 100 ml blood flow divided

by the CO capacity) x 100

↑ AC [COHb] = (0.63/20.1) x 100 = 3.15% sat.

**Calculation: the End-AC [COHb]:** using the ↑ AC[COHb] due to CO uptake 3.15%

sat., and an Inlet-[COHb] of 20% sat..

**End-AC[COHb] = that resulting from CO uptake + that resulting from the Inlet-**

**[COHb]:**

End-AC[COHb] = 3.15 + 20 = 23.15% sat

**Calculation: the Entry-P_CO_:** using MR = 218

***When the Entry-[COHb] equal to an End-AC-[COHb] was 23.15% sat. (The***

***Inlet-[COHb] was 20% sat..)*:** using an arterial PO_2_ of 100 mmHg, and a [O_2_Hb] of 75.9% sat.

**Entry-P_CO_ = ([COHb] x PO_2_)/(MR x [O_2_Hb])**

Entry-P_CO_ = (23.15 x 100)/(218 x 75.9) = 0.141 mmHg

***When the Entry-[COHb] was 42.68% sat. (The Inlet-[COHb] was 40% sat.)*:**

using a PO_2_ of 100 mmHg, and [O_2_Hb] of 57.2% sat..

**Entry-P_CO_ = ([COHb] x PO_2_)/(MR x [O_2_Hb])**

Entry-P_CO_ = (42.7 x 100)/(218 x 57.2) = 0.342 mmHg

**Calculations: the mPC-P_CO_ of 20% capillaries:** using MT = 123. Note, the [COHb]

used in these calculations are considered to be equal to the Entry-[COHb].

***When the Entry-[COHb] was 23.15% sat. and the mPC-PO_2_ 40 mmHg*:** using

an [O_2_Hb] 63.7% and an Entry-P_CO_ 0.141 mmHg,

**(i). The chem eq P_CO_ =** (23.15 x 40)/(123 x 63.7) = 0.118 mmHg

**(ii). The difference between the Entry-P_CO_ and the chem eq P_CO_:**

0.141 - 0.118 = 0.023 mmHg

**(iii). 20% of this difference:** 0.2 x 0.023 = 0.0046 mmHg

**(iv). mPC-P_CO_ = Entry-P_CO_ minus the 20% value:** mPC-P_CO_ = 0.141 - 0.0046 = 0.135 mmHg

***When the Entry-[COHb] was 42.68% sat. and the mPC-PO_2_ 40 mmHg*:**

using an Entry-P_CO_ 0.344 mmHg and an [O_2_Hb] 54.0% sat.

**(i). The chem eq P_CO_** = (42.68 x 40)/(123 x 55.0) = 0.252 mmHg

**(ii). The (Entry P_CO_ - the chem eq P_CO_):** 0.344 - 0.252 = 0.092 mmHg

(**iii). 20% of this decrease:** P_CO_ = 0.2 x 0.092 = 0.018 mmHg

**(iv). The mPC-P_CO_** = 0.344 - 0.018 = 0.326 mmHg

***When the Entry-[COHb] was 23.15% sat. and the mPC-PO_2_ 30 mmHg*:** using

an [O_2_Hb] 55.7% sat. and an Entry-P_CO_ 0.141 mmHg

**(i). The chem eq P_CO_** = (23.15 x 30)/(123 x 55.7) = 0.101 mmHg

**(ii). The (Entry P_CO_ - the chem eq-P_CO_):** 0.141 - 0.101 = 0.040 mmHg

**(iii). 20% of this decrease:** P_CO_ = 0.2 x 0.040 = 0.008 mmHg

**(iv). The mPC-P_CO_** = 0.141 - 0.008= 0.133 mmHg742

***When the Entry-[COHb] was 23.15% sat. and the mPC-PO_2_ 20 mmHg*:** using

an [O_2_Hb] 38.3% sat., and an Entry-P_CO_ 0.141 mmHg

**(i). The chem eq P_CO_** = (23.15 x 20)/(123 x 38.3) = 0.098 mmHg

**(ii). The (Entry P_CO_ - the chem eq P_CO_):** 0.141 - 0.098 = 0.043 mmHg

**(iii). 20% of this decrease:** P_CO_ = 0.2 x 0.043 = 0.0086 mmHg

**(iv). The mPC-P_CO_** = 0.141 - 0.0086 = 0.132 mmHg

***When the Entry-[COHb] was 42.68% sat. and the mPC- PO_2_ 30 mmHg*:** using

an [O_2_Hb] 55.0% sat., and an Entry-P_CO_ 0.344 mmHg

**(i). The chem eq P_CO_** = (42.68 x 30)/(123 x 55) = 0.189 mmHg

**(ii). The (Entry P_CO_ - the chem eq P_CO_):** 0.344 - 0.189 = 0.155 mmHg

(**iii). 20% of this decrease:** P_CO_ = 0.2 x 0.155= 0.031 mmHg

**(iv)**. **The mPC-P_CO_** = 0.344 - 0.031 = 0.313 mmHg

***When the Entry-[COHb] was 42.68% sat and the mPC- PO_2_ 20 mmHg*:** using

an [O_2_Hb] 39.5% sat., and an Entry-P_CO_ 0.344 mmHg

**(i). The chem eq P_CO_** = (42.68 x 20)/(123 x 39.5) = 0.176 mmHg

(**ii). The (Entry P_CO_ - the chem eq P_CO_):** 0.344 - 0.176 = 0.168 mmHg

**(iii). 20% of this decrease:** P_CO_ = 0.2 x 0.168 = 0.034 mmHg

**(iv). The mPC-P_CO_** = 0.344 - 0.034 = 0.310 mmHg
